# Non-typhoidal *Salmonella* in Nigeria: do outcomes of ‘multisectoral’ surveillance, treatment and control justify the intervention costs?

**DOI:** 10.1080/23144599.2024.2365567

**Published:** 2024-07-14

**Authors:** Abdullahi O. Sanni, Abdurrahman H. Jibril, Olubunmi G. Fasanmi, Oluwawemimo O. Adebowale, Alexander R. Jambalang, Aminu Shittu, Annelize Jonker, Latifah O. Abdulkarim, Folorunso O. Fasina

**Affiliations:** a Department of Veterinary Tropical Diseases, University of Pretoria, Pretoria, South Africa; b Agro-Processing, Productivity Enhancement and Livelihood Improvement Support (APPEALS) Project, Lokoja, Nigeria; c Department of Veterinary Public Health and Preventive Medicine, Faculty of Veterinary Medicine, Usmanu Danfodiyo University, Sokoto, Nigeria; d Department of Veterinary Laboratory Technology, Federal College of Animal Health & Production Technology, Ibadan, Nigeria; e Department of Veterinary Public Health and Preventive Medicine, College of Veterinary Medicine, Federal University of Agriculture, Abeokuta, Nigeria; f Bacterial Research Division, National Veterinary Research Institute, Vom, Nigeria & Department of Veterinary Medicine, Surgery and Radiology, Faculty of Veterinary Medicine, University of Jos, Jos, Nigeria; g Faculty of Veterinary Medicine, University of Ilorin, Ilorin, Nigeria; h Emergency Prevention System for Animal Health, Food and Agriculture Organization of the United Nations, Rome, Italy

**Keywords:** Non-typhoidal *Salmonella*, benefit-cost analysis, infectious disease outbreak, One health, Nigeria

## Abstract

Non-typhoidal salmonellosis (NTS) is significant and an economic burden in Nigeria. To determine whether investment in NTS control is economically justifiable, Outbreak Costing Tool (OCT) was used to estimate the robust funding of public and animal health systems for epidemio-surveillance and control of multisectoral NTS outbreaks in Nigeria. Health, production, and economic data were collected and used to populate the tool for evaluation. The multisectoral NTS burden for the year 2020 in Nigeria was US$ 930,887,379.00. Approximately 4,835 technical officers, and 3,700 non-technical staff (*n* = 8,535) were needed with an investment of >2.2 million work hours. The investment cost for NTS control was US$ 53,854,660.87. The non-labour-related cost was 89.21% of the total intervention costs. The overall intervention’s investment was 374.15% of the estimated national and subnational systems’ annual budget for diarrhoeal diseases, and the outbreak response period attracted the highest costs (53%) of the total intervention. In conclusion, intervention against NTS was beneficial (benefit – cost ratio: 17.29), hence justifying the need for multisectoral surveillance-response against NTS in Nigeria. Complex sectoral silos must give way to coordinated collaborations to optimize benefits; and over-centralization of health interventions’ associated delays must be removed through decentralized sub-national-focused framework that empowers rapid investigation, response, control, data collection, and analyses. It should assist anticipatory planning, and outbreak investigation and reduce critical response time. Anticipatory planning tools, when applied pre-emptively, can benefit budgeting, identify gaps, and assist in the delivery of cost-saving and effective measures against infectious disease.

## 1. Introduction

Non-typhoidal *Salmonella* (NTS) is a bacterial zoonosis with significant foodborne impacts in humans worldwide [1]. It can be transmitted to humans, particularly through the consumption of foods of animal origin, including eggs and poultry meat, as well as through direct contact with animals or their environments, especially for people working in the agriculture industry [2,3]. Specifically, humans are infected with NTS through contamination from poultry products (egg fragments, hatching eggs, chick boxes, fluff and faeces), and partially cooked meat and raw eggs [4,5]. More than 2600 serovars of *Salmonella enterica* have been identified, of which, many can cause human infections. However, non-typhoidal serovars, especially *Enteritidis* and *Typhimurium*, are the most isolated serotypes in human infections [6]. Salmonellosis in humans is commonly characterized by diarrhoea, abdominal cramps, fever and vomiting [7]. Although, most non-typhoidal *Salmonella* infections are associated with self-limiting gastroenteritis, they have the potential to cause fatal infections among infants, young children, older adults, and immunocompromised individuals [8]. Diarrhoeal diseases impact life expectancy negatively, particularly in the poor communities [9]. The majority of non-typhoidal *Salmonella* serovars are pathogenic because of their ability to invade, replicate and survive in human host cells [10].

The global estimates of the burden of NTS varied widely, including an estimate of over 27 million human cases and 200,000 deaths per annum [11,12]; approximately 79 million human cases and over 59,000 deaths annually [4]; and 93.8 million human infections and 155,000 fatalities annually [13]. Furthermore, in a ranked study in the USA, *Salmonella* spp. was the first-ranked foodborne pathogen, with the most significant cost of illness and the quality-adjusted life-year (QALY) losses [14]. Based on previous works, environmental risk factors linked with socioeconomic development may trigger situations of unsafe water and poor sanitation, particularly in the underserved rural and peri-urban areas, with consequent increased disability-adjusted life-years (DALYs) [15,16]. Similarly, in such unsanitary conditions, partially cooked meat and raw animal products can be contaminated by processors’ hands, tools and clothing, and this may end up in the human food chain. The importance of hand hygiene among others in limiting disease transmission has previously been emphasized [17].

In Nigeria, the poultry farm-level prevalence of NTS range from 39.7% to 48.3% and the risk factors for NTS infection of poultry farms in Nigeria have been fully explored [18–21]. Based on a recent meta-analytic study, Nigeria has a burden of prevalence (in humans) of 1.9% (2,732/143,756) for *Salmonella* bacteraemia and 5.7–16.3% (1,967/12,081) for *Salmonella*-associated gastroenteritis [22]. In addition, a total of 53 *Salmonella* serotypes have been identified in humans in the country, including 39 associated with *Salmonella*-bacteraemia, and 31 associated with *Salmonella*-gastroenteritis [22].

In the year 2018, the U.S. Centers for Disease Control and Prevention (CDC) commissioned a study in collaboration with RTI International (formerly Research Triangle Institute). This led to the development of the Outbreak Costing Tool (OCT) that estimates intervention costs and useful for a range of disease outbreak scenarios [23,24]. Based on applied intervention and control costs, which can be implemented for humans and animal-specific disease outbreaks, especially where multisectoral responses are required (e.g. zoonotic disease outbreaks), and with a good understanding of disease burden for such disease, a benefit – cost analysis (BCA) may be integrated. The BCA calculates the monetary ratio of all costs to implement a program or course of action and helps determine whether a course of action is worth investing in, based on the assumed worth of the associated benefits. It differs from a tool like the cost-effectiveness analysis (CEA), which assists in selecting the most cost-effective intervention for a defined health outcome, even when multiple methods of intervention are cost-beneficial [25]. Benefit – cost analysis is particularly useful for decision-makers, health leaders, policymakers and resource allocators and for ranking proposals and budgets in the public and animal health sectors. Considering NTS as a One Health challenge in Nigeria, and with a knowledge of its economic and social costs, the application of a tool like the OCT could assist the Nigerian human, animal and environmental health ministries in pre-emptive planning and budgeting for intervention against NTS. The outcomes may also be potentially adaptable to other countries with related burdens of NTS or similar profiles like Nigeria.

This work was implemented to evaluate whether a One Health intervention against NTS is beneficial or not. We aimed to use the OCT to retrospectively generate a cost estimate for comprehensive epidemio-surveillance (investigation, response and control) associated with an all-year-round outbreak of NTS in Nigeria in 2020. The cost-beneficial justification of interventions was measured against the economic and social burdens of NTS. The outcome of this work should assist the authorities in informed decision to prioritize health spendings by utilizing the envisaged derived benefits to justify investments.

## 2. Materials and methods

### 2.1. Spatio-temporal coverage of outbreaks of non-typhoidal salmonellosis

**Figure 1.**
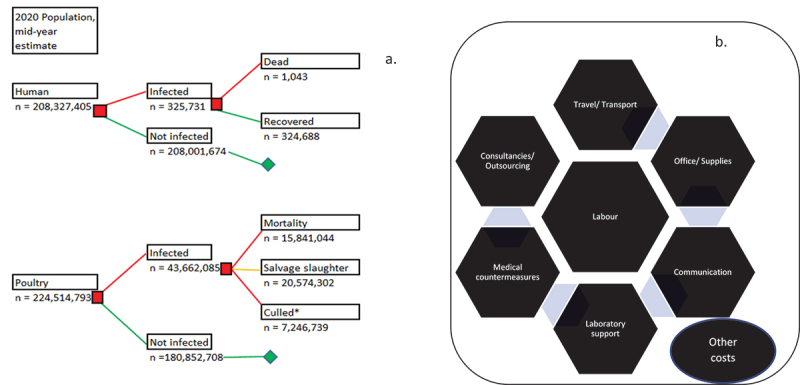
(a) Modelled decision tree population dynamics for non-typhoidal *Salmonella* infection, Nigeria, Jan. – Dec. 2020; (b) Framework for resource category in estimating the cost of surveillance and control in outbreaks of non-typhoidal *Salmonella*, Jan. – Dec. 2020.

Based on the previously validated and published data, we assumed a total of 325,731 human cases of NTS occurred in the year 2020 with a human mortality of 1,043 and a disability-adjusted life year (DALYs) of 37,321 [22, Figure 1]. A total of 188,694 cases (57.9%) occurred among people involved in the poultry value chain while 137,037 (42.1%) occurred among the consumers of poultry and poultry products [26]. In addition, a total of 43,662,085 poultry (chickens) were involved in the 2020 outbreaks from January – December 2020, with 15,841,044 deaths 20,574,302 salvage slaughters, 5,713,152 culls and 1,533,587 unaccounted-for chickens [26, Figure 1]. The total cost of these outbreaks in humans and poultry was a cumulative of US$ 930,887,379.00 [26]. All cases in humans and poultry occurred between the periods, 1 January and 31 December 2020, and all cases were distributed randomly in the country, particularly in the peri-urban and rural areas, and high poultry-dense locations within the country.

Framework for resource category was adapted from Bodenham et al., [24] and steps in a multistate foodborne outbreak investigation from CDC [27]. These steps include: 1). Detect – detect a possible outbreak by monitoring for reported illnesses (salmonellosis) nationwide, 2). Find – define who will be included in the outbreak and look for additional sick people, 3). Generate – generate hypotheses (potential explanations) by interviewing people about what they ate before getting sick, 4). Test – test hypotheses by comparing what sick people ate to what people who are not part of the outbreak ate, 5). Solve – confirm the contaminated food using epidemiologic, laboratory, and traceback information and identify the point of contamination, 6). Control – stop the outbreak by recalling contaminated food, cleaning or closing food facilities, and providing advice to people and businesses, and 7). Decide – Decide the outbreak is over when illnesses stop and the contaminated food is no longer available. Modelled decision tree was adapted from Sanni et al., [26]. *Including 1,533,587 unaccounted infected poultry birds. These may have been consumed unscrupulously or inadvertently. The salvage slaughters also end up in the human food chain largely.

### 2.2. Outbreak management - investigations, responses and controls

According to Ihekweazu et al. [20], salmonellosis is viewed in Nigeria as a moderate zoonosis, ranking low on severity and epidemic potentials but high to moderate on burden of diseases, ability of the health services to control, and socio-economic impacts. Furthermore, salmonellosis, combined with other diarrhoeal diseases, only benefitted from approximately 1.3% of the total mean annual expenditure as a percentage of current health expenditure (CHE) of the Nigerian federal and states’ budgets [28,29]. Hence, NTS will only have partial attribution from the 1.3% funding, an indication that it does not enjoy any prioritized funding or attention as some other rapidly spreading infectious diseases. Basically, there is no budget specifically dedicated for the control of salmonellosis at the federal, state and local government levels except as part of the diarrhoeal diseases. The responsible ministries and government departments or parastatals responsible for salmonellosis management and control include the Federal and States’ Ministries of Agriculture and Livestock Development or Animal Health, and the public and private veterinary clinics (for livestock), and the Federal and States’ Ministries of Health (F/SMoH), Nigeria Center for Disease Prevention and Control (NCDC), the National Primary Health Care Development Agency (NPHCDA), and the primary, secondary and tertiary-level healthcare facilities among others (for humans). Human-level data were also cross-validated with the Surveillance Outbreak Response Management and Analysis System (SORMAS), a tool being used by the Surveillance Unit of the FMOH. In addition, because its fatalities in human is low, the dispositions of most affected individuals with diarrhoeal diseases including NTS were to seek self-therapy at home first, including self-administration of antibiotics, and these individuals would only seek hospitalization in case of increasing severity progressing to fatalities, which is not responsive to home treatment [30,31].

Individual human cases are treated when hospitalization is sought. In cases of aggravated or surge incidences of diarrhoeal diseases in humans at any period of the year, the government’s disease surveillance and control officers at the national and sub-national level would swing into action to investigate, intervene and respond in order to implement control. Such interventions may include the following steps taken in multistate foodborne outbreak investigation [27]: 1). *Detect* - detect a possible outbreak by monitoring for reported illnesses (salmonellosis) nationwide, 2). *Find* - define who will be included in the outbreak and look for additional sick people, 3). *Generate* - generate hypotheses (potential explanations) by interviewing people about what they ate before getting sick, 4). *Test* - test hypotheses by comparing what sick people ate to what people who are not part of the outbreak ate, 5). *Solve* - confirm the contaminated food using epidemiologic, laboratory, and trace back information and identify the point of contamination, 6). *Control* - stop the outbreak by recalling contaminated food, cleaning or closing food facilities, and providing advice to people and businesses, and 7). *Decide* - decide the outbreak is over when illnesses stop, and the contaminated food is no longer available.

For poultry, farmers often vaccinate against fowl typhoid and fowl cholera using commercially available vaccines as preventive protocols. When there are aggravated cases of NTS in poultry, often caused by *Salmonella enterica* serovars *Enteritidis* or *Typhimurium*, farmers typically implement antimicrobial treatment protocols, and if the cases are not resolving, salvage slaughter or culling will supervene. With the above scenarios, the possibility of missing cases in humans and poultry is high.

### 2.3. Questionnaire data collection for outbreak costs, and ethical approval

Data on costs associated with the outbreaks in humans and animals were mined from previous evaluations [26], or collected using validated tools [20, Supplementary Material 1]. A total of 244 field-level datasets on real and estimated costs of intervention for outbreaks of NTS were collected from the government departments and the industry identified in section 2.2 above, from December 2021 to September 2023. A total of 115 datasets (47.1%) originated from the public and animal health officers (medical doctors, veterinary doctors, nurses, pharmacists, consultants, project managers, epidemiologists, surveillance officers and microbiologists). One hundred and twenty (49.2%) datasets came from the laboratory officers (consultants, scientists and technicians), and nine datasets (3.7%) from administrative officers, managers and monitoring and evaluation officers. Approximately 78.7% (*n* = 192) of the responses were obtained through physical face-to-face questionnaire surveys, and only 21.3% (*n* = 52) were obtained through an online survey. Officers and industry stakeholders with knowledge of costs associated with the outbreak were selected as key informants. For ethical consideration, an ethical approval number REC142-22 was obtained from the University of Pretoria, and informed consent was obtained from respondents prior to their involvement in the study. Respondents completed a structured questionnaire pertaining to one or more of the seven OCT independent cost categories: labour, office materials and equipment, travel and transport, communication, laboratory support, medical counter-measure, and consultancies (Supplementary Material 1; Figure 1b). Each cost category questionnaire was designed to generate responses suitable for filling the OCT tool by cost category. When a respondent did not have enough insight or knowledge on specific aspects of a cost category, either the respondent conferred with a colleague for further information or suggested the name of a knowledgeable colleague (snowball) who could complete the remaining cost category fields, and such individual was approached for participation [24]. Questionnaire responses and missing data were cross verified by additional government officials where possible to generate more robust cost estimates and reduce questionnaire bias [32]. This cross-verification of sub-national data was conducted by additional key-informant questionnaire administration, which occurred at national level (Supplementary Material 1).

### 2.4. Integration of outbreak costing tool (oct) to determine costs of intervention, scenario analysis and benefit - cost ratio

In the context of this study, “**multisectoral**” is defined within the context of One Health, wherein collaborative engagement is conducted among multiple discipline and sectors, with a view to ultimately integrate transdisciplinary approach in their working environment (local, regionally, and nationally), with the goal of achieving optimal health outcomes recognizing the interconnection between people, animals, plants, and their shared environment. Such engagements include co-planning, co-working, co-funding and co-implementation in the field [20,24,26]. All costs for disease burdens were retrieved from a previous study [26]. Costing for the investigation, response and control against NTS in Nigeria for the year 2020 was performed using the OCT. The OCT offered a standardized, Excel-based approach to recording and summarizing outbreak costing data [24]. Multisectoral costs were integrated by direct entry of questionnaire-sourced information from multiple sectors into the OCT spreadsheet (Supplementary Material 2); however, we did not break down these costs per each sector based on the protocol of the OCT. All seven cost-related categories were entered comprehensively (Supplementary Material 2 and Supplementary Table 3).

In each category, the individual items, the quantity and cumulative cost per budget line, and the percentage cost for each item were entered in relation to three pre-defined categorization of timelines for outbreak investigation and management: 1) the initial response period (i.e. preparation, outbreak verification, outbreak diagnosis, case verification, case diagnosis, case definition construction, case recording, epidemiology description, hypothesis development, hypothesis evaluation and finalization, and reconciling evidence); 2) the outbreak response period (i.e. implementing infection control and prevention measures); and 3) the follow-up and reporting period (i.e. initiating or maintaining surveillance and dissemination of findings). All entries were verified independently by two of the researchers (SOA, and FOF). The results spreadsheet was shared among all the authors for internal quality control and to identify errors. Results were summarized to facilitate data interpretation, draw inferences and determine the implications of outputs. All cost estimates were calculated at the mid-year exchange rate for the year 2020 (US$1 = N380.26 (local currency) at the time of calculation) [33].

With the understanding that the political, health and financial system are dynamic and that there are many competing interests for limited funds, we used a separate scenario analysis Excel spreadsheet (Supplementary Material 4), and estimated the changes in the impact of interventions and benefit-cost ratios for five scenarios targeting some of the most elaborate cost categories.

Using the total costs of the interventions, and the overall economic and social costs of the burden of NTS, we calculated the benefit–cost ratio (BCR) as follows: *Benefit – Cost Ratio (BCR) of intervention against NTS = (Annual economic and social burdens of diseases ÷ Annual cost of intervention)*.

Where: Annual burden of costs of diseases = US$ 930,887,379.00 [26], and Annual cost of intervention was calculated from the current analysis.

## 3. Results

**Figure 2.**
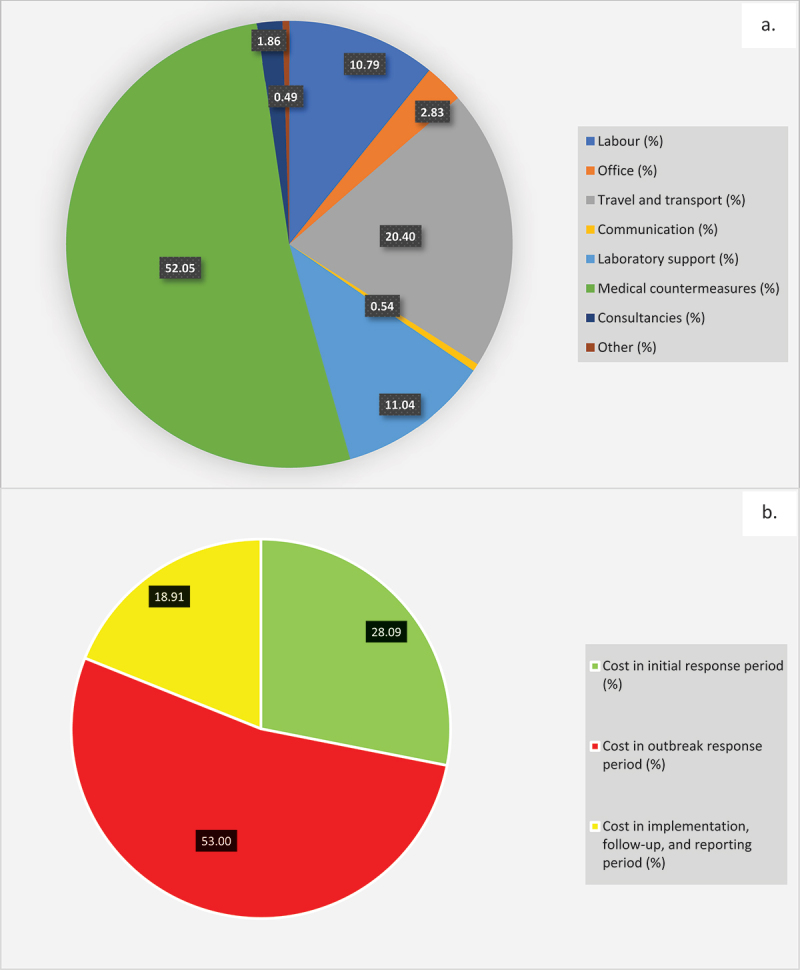
(a) Percentage resource category cost of surveillance and control in outbreaks of non-typhoidal *Salmonella*, Jan. – Dec. 2020; (b) Percentage periodic-based distribution of intervention cost for non-typhoidal salmonellosis outbreak, Nigeria, Jan. – Dec. 2020.

Based on the analyses, an annual effective One Health intervention covering surveillance, management and control of NTS in poultry, and intervention and course of antibiotic treatment in humans in Nigeria will involve at the minimum, approximately 4,835 technical officers and 3,700 non-technical staff (*n* = 8,535), with investment of over 2.2 million work hours at a total cost of US$ 53854,660.87 across the 774 local governments areas of Nigeria (Table 1, Supplementary Material 2). The labour-related cost was US$ 5,811,976.02 (10.79%) of the total intervention cost and the non-labour cost was US$ 48042,684.85 (89.21%). The non-labour cost is subdivided into various categories (see Table 1), with major costs going into medical counter-measure, travels and transports, and laboratory supports (Table 1, Figure 2).

**Table 1.** Summary of outbreaks and intervention cost for non-typhoidal *Salmonella* outbreak, Nigeria, 2020.

Statistics	Value	Unit
Length of outbreak (days)	365	Days
Number of regions affected	37	Number
Number of human cases	325,731*	Number
Number of human deaths	1,043*	Number
Nigerian human population (Midyear, 2020)	208,327,405*	Number
Number of animal cases	43,662,085*	Number
Number of animal deaths	15,841,044*	Number
Nigerian poultry population (Midyear, 2020)	224,326,708*	Number
Non-typhoidal disease burden and social costs	930,887,379.00*	US$
Total labour hours associated with outbreak	2,271,360	Hours
Total intervention cost	**53,854,660.87**	US$
Labour	**5,811,976.02**	US$
Non-labour	**48,042,684.85**	US$
Office	1,524,612.05	US$
Travels and transport	10,987,219.27	US$
Communication	291,905.54	US$
Laboratory Support	5,944,302.86	US$
Medical countermeasures	28,031,667.17	US$
Consultancies	1,000,000.00	US$
Other costs (Miscellaneous)	262,977.96	US$
Total budget for Diarrhoeal Disease Programme	14,393,777.06^#^	US$
Total intervention cost in percentage budget for Diarrhoeal Disease programme (2020)	374.15	%

*Integrated from previous analysis on burden of NTS in Nigeria for the year 2020 [26]. ^#^The Budget for Diarrhoeal Disease Programme is approximately 1.3% of the total mean annual expenditure as percentage of current health expenditure (CHE) of the Nigerian federal and states’ budgets [23,24]. Approximately US$ 12741,647.80 (88.52%) came from the Public Health Programme on Diarrhoeal Diseases and only US$ 1,652,129.26 (11.48%) came from the related Animal Health Programme. The exchange rate at the time of the analysis was Naira 380.26 = US$ 1 (Midyear value, 2020). All cost categories of total expenditure were computed in Nigerian Naira and converted to US$.

Overall, the total intervention cost was 374.15% of the estimated annual budget for the national and subnational systems. Incidentally, the estimated livestock health budget contributed a paltry 11.48% compared to 88.52% contribution from the public health programme on Diarrhoeal Diseases (Table 1).

Comparing the clustered periods of outbreaks, the investment cost during the outbreak response period (53%) was higher than those in the preparedness and initial response period (28.09%) and those spent in the implementation, follow-up, and reporting period (18.91%) (Table 2, Figure 2). Basically, a total of US$ 28541,285.02 was spent between the labour and non-labour costs for implementing treatment, control and prevention measures following outbreaks (Table 3, Figure 2).

**Table 2.** Periodic-based intervention cost category for non-typhoidal *Salmonella* outbreak, Nigeria, 2020.

Category	Overall Cost (US$)	Cost in initial response period (US$)	Cost in outbreak response period (US$)	Cost in implementation, follow-up, and reporting period (US$)
Labor	5,811,976.02	2,982,960.61	2,272,909.59	556,105.82
Nonlabor				
Office	1,524,612.05	880,928.30	434,377.54	209,306.21
Travel and transport	10,987,219.27	1,660,337.66	7,234,628.94	2,092,252.67
Communication	291,905.54	0.00	262,714.98	29,190.55
Laboratory support	5,944,302.86	3,523,277.22	1,479,438.19	941,587.44
Medical countermeasures	28,031,667.17	5,307,424.07	16,595,524.59	6,128,718.51
Consultancies	1,000,000.00	693,000.00	156,500.00	150,500.00
Other	262,977.96	78,893.39	105,191.18	78,893.39
Total Intervention cost for NTS, 2020	**53,854,660.87**	15,126,821.26	28,541,285.02	10,186,554.59
Total intervention cost as a fraction of the budget for Diarrhoeal Disease programme (2020)	3.7415239	1.050927855	1.982890586	0.707705458

**Table 3.** Activity-based intervention cost category for non-typhoidal *Salmonella* outbreak, Nigeria, 2020.

Period	Activity	Cost (US$)	
		Labour	Non-labour
Initial response	Prepare for field work	445,661.55	2,724,103.79
	Establish and verify the existence of an outbreak	397,384.89	2,846,042.87
	Verify the diagnosis	447,128.23	3,432,293.38
	Construct a working case definition	217,663.13	353,076.63
	Find cases systematically and record information	243,302.00	710,951.65
	Perform descriptive epidemiology	182,253.88	839,039.01
	Develop hypothesis	239,177.04	247,521.19
	Evaluate hypothesis epidemiologically	201,986.17	294,338.16
	Reconsider, refine, and re-evaluate hypothesis	176,298.53	263,831.10
	Compare and reconcile with laboratory and/or environmental studies	432,105.19	432,662.86
Outbreak response	Implement treatment, control and prevention measures	2,272,909.59	26,268,375.43
Follow up and reporting	Initiate or maintain surveillance to determine whether the prevention and control measures are working	90,632.67	7,666,217.76
	Write an outbreak investigation report and disseminate findings appropriately	465,473.15	1,964,231.00
Total intervention cost per category		5,811,976.02	48,042,684.85
Total intervention cost as fraction of category allocated budget (%)		48.35	2,024.10

1. Benefit – Cost Ratio (BCR) of intervention = Annual burden of costs of diseases ÷ Annual cost of intervention
2. Where: Annual burden of costs of diseases = US$ 930,887,379.00 [22], and annual cost of intervention = US$ 53854,660.87.
3. BCR = US$ (930,887,379.00 ÷ 53,854,660.87) = **17.29** (Table 4).

**Table 4.** Scenario Analysis of variation in Intervention costs and impacts on Benefit–Cost Ratio of Programme Intervention against NTS, 2020.

Resource Category	Original	Scenario 1	Scenario 2	Scenario 3	Scenario 4	Scenario 5
Labour	5,811,976.02	8,136,766.43	9,299,161.63	9,299,161.63	5,811,976.02	5,811,976.02
Travel/transport	10,987,219.27	6,592,331.56	6,592,331.56	6,592,331.56	13,184,663.12	15,382,106.98
Office supplies	1,524,612.05	1,524,612.05	1,905,765.06	1,905,765.06	1,753,303.86	1,524,612.05
Communications	291,905.54	291,905.54	291,905.54	291,905.54	291,905.54	321,096.09
Laboratory support	5,944,302.86	8,322,024.00	8,322,024.00	8,916,454.29	7,133,163.43	5,944,302.86
Medical countermeasures	28,031,667.17	28,031,667.17	25,228,500.45	28,031,667.17	23,826,917.09	28,031,667.17
Consultancies/outsourcing	1,000,000.00	1,000,000.00	1,000,000.00	1,500,000.00	1,000,000.00	1,000,000.00
Others/miscellaneous	262,977.96	262,977.96	262,977.96	262,977.96	262,977.96	262,977.96
**Total Program cost**	**53,854,660.87**	**54,162,284.71**	**52,902,666.21**	**56,800,263.22**	**53,264,907.03**	**58,278,739.13**
**Percentage of original**	**100.00**	**100.57**	**98.23**	**105.47**	**98.90**	**108.21**
	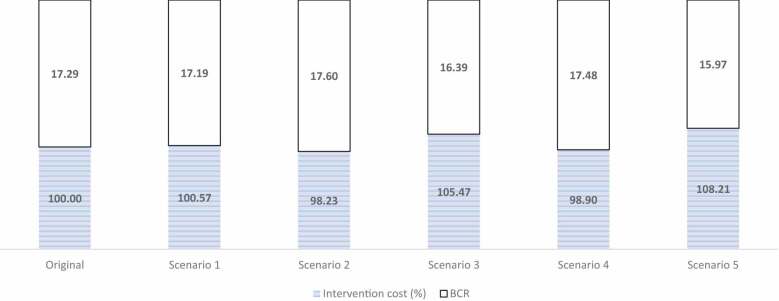					

**Scenario 1**: By increasing of labour and laboratory support costs each by 40% while decreasing travel and transport costs by 40%, the new total cost of the surveillance programme against NTS will be 100.57% of original cost, thus reducing the BCR marginally to 17.19; **Scenario 2**: Even if labour costs are increased by 60%, laboratory support costs are increased by 40% and office supplies costs are increased by 25%, while decreasing travel and transport costs by 40% and medical countermeasure costs by 10%, the new total cost of the surveillance programme against NTS will be 98.23% of original cost, thus increasing the BCR marginally to 17.6; **Scenario 3**: By adding an additional 20% to the laboratory support costs to make a new cumulative of 60%, and with an additional 50% increase in the costs associated with consultancies/outsourcing, added to scenario 2, the new total cost of the surveillance programme against NTS will be 105.47% of original cost, thus reducing the BCR marginally to 16.39; **Scenario 4**: If the travel and transport costs increase by 20%, and laboratory support costs increase by 20% while office supplies costs increase by 15% and medical countermeasure costs reduce by 15%, the new total cost of the surveillance programme against NTS will be 98.90% of original cost, thus increasing the BCR to 17.48; **Scenario 5**: Finally, if the travel and transport costs increase by 40%, and communication costs increase by a marginal 10% while all other parameters remain the same, the new total cost of the surveillance programme against NTS will be 108.21% of original cost, thus decreasing the BCR to 15.97.

By increasing labour and laboratory support costs each by 40% while decreasing travel and transport costs by 40%, the new total cost of the surveillance programme against NTS will be 100.57% of original cost, thus reducing the BCR marginally to 17.19. Even if labour costs are increased by 60%, laboratory support costs are increased by 40% and office supplies costs are increased by 25%, while decreasing travel and transport costs by 40% and medical counter-measure costs by 10%, the new total cost of the surveillance programme against NTS will be 98.23% of original cost, thus increasing the BCR marginally to 17.6; By adding an additional 20% to the laboratory support costs to make a new cumulative of 60%, and with an additional 50% increase in the costs associated with consultancies/outsourcing, added to scenario 2, the new total cost of the surveillance programme against NTS will be 105.47% of original cost, thus reducing the BCR marginally to 16.39 (Table 4).

Furthermore, if the travel and transport costs increase by 20%, and laboratory support costs increase by 20% while office supplies costs increase by 15% and medical counter-measure costs reduce by 15%, the new total cost of the surveillance programme against NTS will be 98.90% of original cost, thus increasing the BCR to 17.48. Finally, if the travel and transport costs increase by 40%, and communication costs increase by a marginal 10% while all other parameters remain the same, the new total cost of the surveillance programme against NTS will be 108.21% of original cost, thus decreasing the BCR to 15.97 (Table 4).

## 4. Discussion

In this analysis, first, we aimed to estimate the costs of multisectoral (human – animal) investigation and response activities associated with a year outbreak of non-typhoidal salmonellosis in Nigeria for the year 2020. In addition, we conducted a benefit – cost analysis of the intervention to determine whether it is worth investing in the epidemio-surveillance, prevention and control of NTS in Nigeria and evaluated different scenarios considering the multisectoral competing interests for limited available funds, other health priorities and unplanned but emergent needs of the country. The OCT estimated the comprehensive costs of interventions against NTS in humans and poultry in Nigeria for the year 2020 (US$ 53854,660.87) and categorized the cost into various subheads and stages of outbreak investigation and response periods. Such division becomes necessary in order to prioritize anticipatory planning, budgeting and identify funding gaps while providing effective responses against infectious diseases [24,34]. As previously suggested by Bodenham and colleagues [24], the OCT is a utility tool that can be used at multiple tiers and levels – for example, at different government ministries, departments and parastatals, and can be coordinated with other tools like the *Multisectoral Coordination Mechanism Operational Tool* for annual coordination and costing for all anticipated One Health activities in the country [35]. Although there are other existing tools [36,37], which can be used for the estimation of burdens of diseases nationally or globally, the choice of the OCT in this study was data driven and based on previous experience and its utility.

First, we observed significant under-resourcing and under-provisioning for the overall Diarrhoeal Disease Programme, salmonellosis and more specifically, the NTS intervention in humans and animals. For instance, based on our estimates, the budget needed to perform efficiently an annual intervention against NTS was 274.15% above the allocated budget for the year 2020. Not surprisingly, salmonellosis is not high-prioritized foodborne zoonoses in Nigeria despite its ranking as high to moderate on the burden of diseases, the ability of the health services to control it, and its socio-economic impacts [20]. Secondly, budget distribution among the subheads (personnel, overhead and capital) in Nigeria weighs heavily in favour of personnel. Our analysis indicated that while the personnel may utilize less than 50% of its resources, the non-labour category utilized over 2000% of its allocated resources, a pointer that there may be a need to relook at the whole budgeting process to allocate more to activities and possibly rationalize the workforce where necessary. Worse still, the estimated livestock health budget contributed a paltry 11.48% of the Diarrhoeal Disease Programme for the year 2020, an indication that much less allocation will be directed at non-typhoidal salmonellosis’ surveillance, management and control. In this wise, there is bound to be ineffective Veterinary Services to tackle diseases like NTS at both national and subnational levels [24,34]. It should be noted that poultry remains one of the major sources of NTS in humans, and the Nigerian poultry value chain and informal trade enables random nationwide distribution of untested poultry and its products, with risk of long-distance transmission of NTS within Nigeria. It is expected that mitigating NTS risks in poultry will significantly reduce the social and economic burdens of NTS in humans. Thus, we advocate more investment in vaccination against fowl typhoid in poultry, and in effective surveillance, monitoring and control of salmonellosis in the poultry value chain – in particular, at the hatcheries, day-old-chicks, eggs and poultry meat distribution networks to mitigate NTS impacts. Recently, the World Bank Group has shown that investment in One Health Systems based on disease prevalence will generate expected returns and prevent pandemics by half or entirely [38], with similar studies originating from the Global Burden of Disease 2021 Health Financing Collaborator Network [39]. Such investment scenarios could be facilitated or reviewed through tabletop or limited simulation exercises to test the likely effectiveness of such investment.

The overall estimated intervention costs cover the entirety of the outbreak year from 1^st^ January until 31^st^ December 2020 NaN Invalid Date considering the burden of infection and deaths in human and animals, however, the estimate is for planning purposes since the dynamics of disease outbreaks is absolutely unpredictable and may respond differently under many circumstances. Distilling this further, the cost associated with the period of initial response was 28.09% of the total costs, while those related to outbreak response and follow-up and reporting were 53.00% and 18.91%, respectively. This is similar to cost distribution for scenario analysis for anthrax intervention in Tanzania in 2018–2019 [24]. Perhaps, an investment in preparedness and initial response period (pre-outbreak periods otherwise known as peacetime and alert period) will aid early detection, limit the scale of outbreaks thus limiting disease burdens and the eventual impact and costs of managing the outbreaks as indicated in the scenario analysis [40]. The bulk of the costs invested in the annual management of NTS in Nigeria are embedded in the outbreak period’s medical counter-measure, travel and transport, laboratory and labour. Hence, every effort aimed at reducing the unit costs in these categories will have overall impacts in increasing the benefit-cost, reducing the associated disease burdens and the costs of intervention.

Understanding the distribution of these estimated costs associated with different NTS outbreak and response periods and categories can assist in effective budgeting and planning for future outbreaks and possibly has lateral positive effects in planning for other diseases. Bodenham and colleagues [24], have earlier stressed the benefit of such planning. Whereas such plans must be innovatively engaged by the technical and non-technical officers, it can also be used with the planners and policymakers for advocacy both at the national and subnational levels.

The implications of this study are as follows: 1) It provides the platform for policy prioritization, wherein policymakers make informed decision on the economic viability and potential returns on investment in *Salmonella* infection control measures; 2) It should assist in resource allocation using cost-beneficial methods; 3) It has the capacity to assist in formulating effective public health interventions, including the design and implementation of public health policies aimed at reducing the burden of *Salmonella* infections in Nigeria; 4) It can inform the design and implementation of disease surveillance and monitoring systems for tracking the prevalence and impact of *Salmonella* infections in humans and animals; and 5) This outcome can facilitate closer interactions among the cross-sectoral partners in health, policy and planning.

### 4.1. Limitations of the study

The major limitation of our investigation was the use of small number of participants to obtain the cost data, as this may have influenced the cost estimates generated. It should be noted that the tool was applied for the scenarios for the year 2020, approximately 2 years from the hypothetical outbreak events, because the calculations on the burdens of the disease had been set for the year 2020; this may have subjected the study to a degree of recall bias. We however cross-validated several pieces of information obtained from key informants and institutions. Where some degree of inconsistency exists in qualitative information, we checked official record or other information sources.

Though cost analyses for infectious disease outbreaks is challenging due to data scarcity of cost data, and dearth of records or a single repository where all the data can be obtained [41], its outputs and outcomes are vital for pushing boundaries and getting supports for investment in public and animal health. The availability and use of simple, fast and adaptable tools, such as the OCT, may assist in bridging these data gaps and building capacity in this area. Overall, the proposed intervention in this study was 17.29 cost beneficial for NTS and different scenarios presented with different positive benefit–cost ratio. Hence, investment in diarrhoeal disease programme and foodborne zoonoses like NTS will be at least 16 folds worthwhile with benefits for other health programmes since many labour and non-labour resources will be shared across platforms.

In view of the burden of costs associated with medical counter-measure and travels and transport and considering the many competing yet important interests for the depleting resources in many low-and-middle-income countries (LMICs), a re-prioritization of budgeting and allocation of scarce resources are desirable using innovative approach. For instance, highly trained and very competent sub-national veterinary workforce will reduce heavy dependence on national officers, and shorten the critical response time to intervention, the burden of diseases and ultimately the heavy costs associated with travel [42]. However, such trained manpower must be capacitated with resources (surveillance materials, tools, consumables and equipment) to carry out their mandate, with the consequent effective utilization of sub-national officers. It may also improve the utilization of national officers as these will have more time to focus on planning, coordination and provision of overall backup services (surge capacity) to sub-national systems where needed. Introduction and use of electronic assistance (tools, apps, artificial intelligence, etc.) for reporting, coordination, response and control may improve the four-way linkages among veterinary and public health’s field and laboratory workforce at both national and sub-national levels [43,44]. It may be important to consider zonal or regional logistic supplies or stores for public health and veterinary services, to eliminate long waiting time and aid easy access to logistics, supplies and consumables that supports epidemio-surveillance and monitoring. While such coordination and lead distributions may be central, utilization and unhindered access should be subnational once any significant health event occurs or at short notice [45].

Furthermore, in a realistic world, disease situation, financial and political dynamics could change rapidly; hence, we made a number of assumptions as outlined in work and premise on the stable political economy. It is hoped that the situation remains as suggested as any significant change may affect the outputs and outcomes of the analyses. Considering this dynamic, we suggested some scenarios and presented a supplemental material that may guide scenario planning. In addition, the salary category for labour is subjected to some subjectivity either because salaries are personal and individuals do not want to talk about their salaries, or the total emoluments per each intervention may be difficult to predict since the length and scope of outbreaks may differ. To adjust for this, we utilized the admin and finance-level information to benchmark personal-level information and use mean (or median) figures where applicable and we used subject matters specialists’ opinions to determine lengths and potential scopes of NTS.

## 5. Conclusions

Multisectoral investigation and response against NTS in Nigeria can benefit from health re-focusing and re-prioritization. However, it may also become complex due to current sectoral silos, uneven sectoral financing, coordination challenges worsened by delays associated with over-centralization of public and animal health interventions. A decentralized framework with sub-national focus and empowerment for rapid investigation, response and control is necessary. Such system should be used for collecting and analysing useful cost and epidemio-surveillance data to aid understanding of under-estimated outbreaks like NTS. Assisted anticipatory planning, and early outbreak investigation will reduce critical response time, and tools like OCT or those comparable to it, if applied pre-emptively, can benefit budget planning, identify gaps in current surveillance methods, and assist in proposition of cost saving but effective measures against infectious disease.

## Supplementary Material

IJVMS_Supplementary Material 1-4 (1)...docx
