# Supplementary material for: Non-typhoidal Salmonella in Nigeria: do outcomes of ‘multisectoral’ surveillance, treatment and control justify the intervention costs?
Source: Int J Vet Sci Med. 2024 Jul 14;12(1):48–59. doi: 10.1080/23144599.2024.2365567 (PMC11249158; doi:10.1080/23144599.2024.2365567)
Supplement: IJVMS_Supplementary Material 1-4 (1)...docx [file TVSM_A_2365567_SM0123.docx]

##### **Supplementary Material 1**

##### APPENDIX 1: CONSENT FORM

**PARTICIPANT INFORMATION SHEET**

_______/________ 202

Title: Eco-Epidemiology and Microbiological Evaluation of Poultry Salmonellosis in North Central Nigeria, and its Socio-economics and Public Health Impacts

Lead Researcher/Student Name: Sanni Abdullahi OZOMATA

Student Number: 22959590

University: University Of Pretoria, South Africa

Faculty: Veterinary Science

Department: Veterinary Tropical Diseases

Programme: PhD (Veterinary Tropical Diseases)

Candidate Physical address: House 57, Aviation Housing Estate, F.C.T Abuja, Nigeria.

Email address: drsao.epidem@gmail.com

Phone number: +234 803 608 0269

*Under the supervision of the following persons*:

Supervisor Prof. Folorunso O. FASINA (daydupe2003@yahoo.co.uk)

Co-Supervisor Dr. Annelize JONKER (annelize.jonker@up.ac.za)

**Dear Prospective Participant**

My name is Abdullahi Ozomata SANNI, I am doing research under the supervision of Prof. Folorunso O. FASINA, an Extraordinary Professor in the Department of Vet. Tropical Diseases at the University of Pretoria. My study will lead to the award of PhD (Vet Sc.) Degree from the University of Pretoria. We are inviting you to participate in a study under the broad title “**Eco-Epidemiology and Microbiological Evaluation of Poultry Salmonellosis in North Central Nigeria, and its Socio-economics and Public Health Impacts**.” This specific study is aimed at understanding the **Economic and Social Burdens of Non-Typhoidal Salmonella Infections**.

**WHAT IS THE PURPOSE OF THE STUDY?**

The aim of this study is understanding the Economic and Social Burdens of Non-Typhoidal Salmonella Infections.

**WHY AM I BEING INVITED TO PARTICIPATE?**

You have been selected as a stakeholder in the industry through direct identification, recommendation or nomination from your area of expertise or contributions.

The totality of the study has been discussed with the authorities of the Federal Ministry of Agriculture and Rural Development, Abuja Nigeria. Permission has been obtained and the total number of participants in this study will be dependent on when the saturation point is reached because we are using industry and publicly available data and participants are recruited through snowballing method.

**WHAT IS THE NATURE OF MY PARTICIPATION IN THIS STUDY?**

The study involves the use of questionnaires, which will be administered using face to face method, or through Google Forms (<https://docs.google.com/forms/d/e/1FAIpQLSefH1i8YASvewU1y1x-OS0sgyuvWJnOuaECXKH9ReLV4YaYZw/viewform?vc=0&c=0&w=1&flr=0>). The expected duration of participation and the time needed to collect data is approximately 30 – 60 minutes per participant, depending on areas that concern each participant.

**CAN I WITHDRAW FROM THIS STUDY EVEN AFTER HAVING AGREED TO PARTICIPATE?**

Participation in this research is entirely voluntary. It is participant’s choice whether to participate or not. Participant may change their mind later and stop participating even if they agreed earlier.

**WHAT ARE THE POTENTIAL BENEFITS OF TAKING PART IN THIS STUDY?**

This study should make empirical data available, which should assist implementation research, decision science, inform future government policy and benefit the poultry sector of the agricultural industry.

**ARE THEIR ANY NEGATIVE CONSEQUENCES FOR ME IF I PARTICIPATE IN THE RESEARCH PROJECT?**

There are no foreseeable risks of harm or side effects to you by participating in this study. The only inconvenience to you will be your valuable time that you will sacrifice answering the questions in the questionnaire.

**WILL THE INFORMATION THAT I CONVEY TO THE RESEARCHER AND MY IDENTITY BE KEPT CONFIDENTIAL?**

All the answers from the participants to be used will be viewed as strictly confidential, and only members of the research team will have access to the information. No data published in dissertations and journals will contain any information about name, address and picture. Your anonymity is therefore ensured.

**HOW WILL THE RESEARCHER(S) PROTECT THE SECURITY OF DATA?**

Questionnaires will be kept under lock and key until the capturing has been completed. Only the researcher will have access to the questionnaires. The raw data will be captured in Microsoft Excel Spread Sheet and stored with on the researcher’s computer with a protective password and an external drive as a backup. After the study had been completed the data will be kept for a period of 3 years, but will not be used in any further studies.

**WILL I RECEIVE PAYMENT OR ANY INCENTIVES FOR PARTICIPATING IN THIS STUDY?**

Participating in this study is voluntary and participants are not entitled to any payment.

**HAS THE STUDY RECEIVED ETHICS APPROVAL?**

Yes.

**HOW WILL I BE INFORMED OF THE FINDINGS/RESULTS OF THE RESEARCH?**

If you would like to be informed of the final research findings, please contact Dr Abdullahi Sanni with the email and phone numbers displayed on the first page of this document. The findings are accessible from the time of publication in the journal that accept the manuscript for peer-review publication, and also permanently in the associated PhD Thesis of Abdullahi Ozomata SANNI at the University of Pretoria.

Should you have concerns about the way in which the research has been conducted, you may contact Prof. Folorunso O. FASINA, e-mail: [folorunso.fasina@fao.org](mailto:folorunso.fasina@fao.org).

Thank you for taking time to read this information sheet and for participating in this study.

If you agree with the above content, you will sign or thumbprint the following, or use digital signature (for online Google Form) and we will now proceed with the interview.

| Participant name: |  |
| --- | --- |
| Participant signature and date |  |

Regards
Dr. A. O. Sanni

Department of Veterinary Tropical Diseases, University of Pretoria

E-mail: drsao.epidem@gmail.com

Phone number: +234 803 608 0269

**Supplementary Material 1. Questionnaires to target specific cost head for the Outbreak Costing Tool**

**Costing for hypothetical non-typhoidal salmonella outbreak, 2020, Nigeria**

Specific Labour Costs

**Respondent details**

Name:

Position:

Email:

Phone:

Costing for hypothetical non-typhoidal salmonella outbreak, 2020, Nigeria

**Section 1 of 7 - Specific Labour Costs**

*Please complete any monetary questions in* ***Nigerian Naira***

*If any section below is not applicable to the current outbreak, please write N/A*

*The following 4 questions are to be answered for each of the listed job titles below (where applicable) and for any additional job titles absent from the list:*

**1 - Average monthly salary (including benefits)**

**2 - Typical work hours in a month (in an average month)**

**3 - Average number of hours spent in outbreak investigation & response activities (over duration of the outbreak)**

**4 - Number of staff in this particular role that worked on outbreak investigation & response activities**

Job title 1: **Epidemiologist**

1 –

2 –

3 –

4 –

Job title 2: **Public Health Specialist**

1 –

2 –

3 –

4 –

Job title 3: **Medical Specialist**

1 –

2 –

3 –

4 –

Job title 4: **Nurse**

1 –

2 –

3 –

4 –

Job title 5: **Pharmacist**

1 –

2 –

3 –

4 –

Job title 6: **Lab Technician**

1 –

2 –

3 –

4 –

Job title 7: **Data analyst**

1 –

2 –

3 –

4 –

Job title 8: **Community engagement specialist**

1 –

2 –

3 –

4 –

Job title 9: **Project Manager**

1 –

2 –

3 –

4 –

Job title 10: **Director of Outbreak Response**

1 –

2 –

3 –

4 –

Job title 11: **Veterinarian**

1 –

2 –

3 –

4 –

Job title 12: **Other(specify) __________________**

1 –

2 –

3 –

4 –

Job title 13: **Other(specify) __________________**

1 –

2 –

3 –

4

NB - There is a more complex addition to this section, covering the percentage of value hours and how they were distributed for each individual job title across each stage of the outbreak investigation. A percentage of total expenditure form for this section is to be completed after this initial section is complete.

**Costing for hypothetical non-typhoidal salmonella outbreak, 2020, Nigeria**

**Specific labour costs questionnaire: Percentage of total expenditure form**

*For your job role, please indicated below what percentage of your total value hours was incurred during each period of the outbreak (each applicable row should total 100%):*

| **JOB TITLE** | **INITIAL RESPONSE PERIOD: *% of value hours reported***  Including the following activities:   - Prepare - Verify outbreak - Verify diagnosis - Construct case definition - Record cases - Perform descriptive epidemiology - Develop hypothesis - Evaluate hypothesis - Refine hypothesis - Reconcile evidence | **OUTBREAK RESPONSE PERIOD: *% of value hours reported***  Including:   - Implement control & prevention measures | **IMPLEMENTATION FOLLOW UP AND REPORTING PERIOD: *% of value hours reported***  Including:   - Initiate or maintain surveillance - Disseminate findings | **TOTAL (%)**  **(each individual row should total 100%)** |
| --- | --- | --- | --- | --- |
|  |  |  |  |  |
|  |  |  |  |  |
|  |  |  |  |  |
|  |  |  |  |  |
|  |  |  |  |  |

**Costing for hypothetical non-typhoidal salmonella outbreak, 2020, Nigeria**

Office Materials & Equipment Costs

**Respondent details**

Name:

Position:

Email:

Phone:

Costing for hypothetical non-typhoidal salmonella outbreak, 2020, Nigeria

**Section 2 of 7 – Office Material & Equipment Costs**

*Please complete any monetary questions in* ***Nigerian Naira***

*If any section below is not applicable to the current outbreak, please write N/A*

*The following 2 questions are to be answered for each of the listed office consumables below (where applicable), and for any additional office-related items absent from the list:*

**1 - Quantity used**

**2 - Total expenditure on item to support outbreak investigation and response activities**

Office supplies 1: **Stationeries**

1 –

2 –

Office supplies 2: **Printing/copies**

1 –

2 –

Building & Office Equip Rental 1:

**Rented building space**

1 – N/A

2 –

Building & Office Equip Rental 2:

**Rented equipment**

1 – N/A

2 –

Building & Office Equip Rental 3:

**Rented furniture**

1 – N/A

2 –

Telecommunications/Electronics 1:

**Internet/Wifi**

1 – N/A

2 –

Telecommunications/Electronics 2:

**Mobile phone data**

1 – N/A

2 –

Telecommunications/Electronics 3:

**Specialty software**

1 – N/A

2 –

Telecommunications/Electronics 4:

**Mobile phones**

1 –

2 –

Telecommunications/Electronics 5:

**Solar panels to charge phones & computers**

1 –

2 –

Telecommunications/Electronics 6:

**GPS**

1 –

2 –

Telecommunications/Electronics 7:

**Mobile Hotspots**

1 –

2 –

Other 1 (specify)**: __________________________**

1 –

2 –

Other 2 (specify): **__________________________**

1 –

2 –

*Please identify any office staff positions directly related to these outbreak investigation and response activities:*

1 Job title:

1 Number of individuals:

2 Job title:

2 Number of individuals:

3 Job title:

3 Number of individuals:

4 Job title:

4 Number of individual

NB - There is a more complex addition to this section, covering the percentage of total expenditure for each item reported and how it is distributed across each individual stage of the outbreak investigation. A percentage of total expenditure form for this section is to be completed after this initial section is complete.

**Costing for hypothetical non-typhoidal salmonella outbreak, 2020, Nigeria**

Travel & Transport Costs

**Respondent details**

Name:

Position:

Email:

Phone:

Costing for hypothetical non-typhoidal salmonella outbreak, 2020, Nigeria

**Section 3 of 7 – Travel & Transport Costs**

*Please complete any monetary questions in* ***Nigerian Naira***

*If any section below is not applicable to the current outbreak, please write N/A*

*The following 2 questions are to be answered for each of the listed travel and transport items below (where applicable), and for any additional travel and transport-related items absent from the list:*

**1 - Quantity used**

**2 - Total expenditure on item to support outbreak investigation and response activities**

Vehicle related costs 1: **Fuel costs**

1 – N/A

2 –

Vehicle related costs 2: **Rented or hired vehicles**

1 –

2 –

Vehicle related costs 3: **Parking (quantity = days)**

1 –

2 –

Vehicle related costs 4: **Purchased vehicles**

1 –

2 –

Vehicle related costs 5: **Maintenance & repair costs**

1 – N/A

2 –

Travel & Lodging 1: **Lodging (quantity = nights)**

1 –

2 –

Travel & Lodging 2: **Per diem expenses (food etc.) (quantity = days)**

1 –

2 –

Travel & Lodging 3: **Airfare for deployed personnel**

1 – N/A

2 –

Travel & Lodging 4: **Taxi & Bus fares**

1 – N/A

2 –

Other 1 (specify)**: __________________________**

1 –

2 –

Other 2 (specify): **__________________________**

1 –

2 –

*Please identify any travel and transport staff positions directly related to these outbreak investigation and response activities:*

1 Job title:

1 Number of individuals:

2 Job title:

2 Number of individuals:

3 Job title:

3 Number of individuals:

4 Job title:

4 Number of individuals:

NB - There is a more complex addition to this section, covering the percentage of total expenditure for each item reported and how it is distributed across each individual stage of the outbreak investigation. A percentage of total expenditure form for this section is to be completed after this initial section is complete.

**Costing for hypothetical non-typhoidal salmonella outbreak, 2020, Nigeria**

Communication Costs

**Respondent details**

Name:

Position:

Email:

Phone:

Costing for hypothetical non-typhoidal salmonella outbreak, 2020, Nigeria

**Section 4 of 7 – Communication Costs**

*Please complete any monetary questions in* ***Nigerian Naira***

*If any section below is not applicable to the current outbreak, please write N/A*

*The following 2 questions are to be answered for each of the listed communication items below (where applicable), and for any additional communication-related items absent from the list:*

**1 - Quantity used**

**2 - Total expenditure on item to support outbreak investigation and response activities**

Outreach/Awareness 1: **Airtime for national radio broadcasts to communicate/warn about outbreak**

1 – N/A

2 –

Outreach/Awareness 2: **Airtime for national television broadcasts to communicate/warn about outbreak**

1 – N/A

2 –

Outreach/Awareness 3: **Outbreak ads in national newspapers**

1 –

2 –

Outreach/Awareness 4: **Airtime for local radio broadcast to communicate/warn about outbreak**

1 – N/A

2 –

Outreach/Awareness 5: **Airtime for local television broadcasts communicate/warn about outbreak**

1 – N/A

2 –

Outreach/Awareness 6: **Ads in local newspapers to communicate/warn about outbreak**

1 –

2 –

Outreach/Awareness 7: **Wall Posters to communicate/warn about outbreak**

1 –

2 –

Outreach/Awareness 8: **T-shirts to raise awareness for outbreaks**

1 –

2 –

Other 1 (specify)**: __________________________**

1 –

2 –

Other 2 (specify): **__________________________**

1 –

2 –

*Please identify any communications staff positions directly related to these outbreak investigation and response activities:*

1 Job title:

1 Number of individuals:

2 Job title:

2 Number of individuals:

3 Job title:

3 Number of individuals:

4 Job title:

4 Number of individuals:

NB - There is a more complex addition to this section, covering the percentage of total expenditure for each item reported and how it is distributed across each individual stage of the outbreak investigation. A percentage of total expenditure form for this section is to be completed after this initial section is complete.

**Costing for hypothetical non-typhoidal salmonella outbreak, 2020, Nigeria**

Laboratory Support Costs

**Respondent details**

Name:

Position:

Email:

Phone:

Costing for hypothetical non-typhoidal salmonella outbreak, 2020, Nigeria

**Section 5 of 7 – Laboratory Support Costs**

*Please complete any monetary questions in* ***Nigerian Naira***

*If any section below is not applicable to the current outbreak, please write N/A*

*The following question is to be answered for the laboratory items listed below (where applicable), and for any additional laboratory-related items absent from the list:*

**1 - Total expenditure on item to support outbreak investigation and response activities**

Specimen testing 1: **Identification of pathogens**

1 –

Specimen testing 2: **Data management**

1 –

Specimen testing 3: **Data analysis and results**

1 –

Specimen testing 4: **Waste management**

1 –

Other 1 (specify)**: __________________________**

1 –

Other 2 (specify): **__________________________**

1 –

*Please identify any laboratory staff positions directly related to these outbreak investigation and response activities:*

1 Job title:

1 Number of individuals:

2 Job title:

2 Number of individuals:

3 Job title:

3 Number of individuals:

4 Job title:

4 Number of individuals:

NB - There is a more complex addition to this section, covering the percentage of total expenditure for each item reported and how it is distributed across each individual stage of the outbreak investigation. A percentage of total expenditure form for this section is to be completed after this initial section is complete.

**Costing for hypothetical non-typhoidal salmonella outbreak, 2020, Nigeria**

Medical Countermeasures Costs

**Respondent details**

Name:

Position:

Email:

Phone:

Costing for hypothetical non-typhoidal salmonella outbreak, 2020, Nigeria

**Section 6 of 7 – Medical Countermeasures (Non-labour) Costs**

*Please complete any monetary questions in* ***Nigerian Naira***

*If any section below is not applicable to the current outbreak, please write N/A*

*The following 2 questions are to be answered for each of the listed medical countermeasures items below (where applicable), and for any additional medical countermeasures-related items absent from the list:*

**1 - Quantity used**

**2 - Total expenditure on item to support outbreak investigation and response activities**

Drugs 1: **Drugs for prevention: Vaccines**

1 –

2 –

Drugs 2: **Antibiotic prophylaxis**

1 –

2 –

Drugs 3: **Additional drugs (specify): ________________________________________**

1 –

2 –

Drugs 4: **Additional drugs (specify): ________________________________________**

1 –

2 –

Drugs 5: **Additional drugs (specify): ________________________________________**

1 –

2 –

Control measures 1: **Quarantine**

1 – N/A

2 –

Control measures 2: **Closing food premises**

1 – N/A

2 –

Control measures 3: **Animal culls**

1 – N/A

2 –

Control measures 4: **Disposal or decontamination of contaminated items**

1 – N/A

2 –

Prevention measures 1: **Water chlorination**

1 – N/A

2 –

Prevention measures 2: **Impregnated bed nets**

1 –

2 –

Other 1 (specify)**: __________________________**

1 –

2 –

Other 2 (specify): **__________________________**

1 –

2 –

*Please identify any medical staff positions directly related to these outbreak investigation and response activities:*

1 Job title:

1 Number of individuals:

2 Job title:

2 Number of individuals:

3 Job title:

3 Number of individuals:

4 Job title:

4 Number of individuals:

5 Job title:

5 Number of individuals:

6 Job title:

6 Number of individuals:

NB - There is a more complex addition to this section, covering the percentage of total expenditure for each item reported and how it is distributed across each individual stage of the outbreak investigation. A percentage of total expenditure form for this section is to be completed after this initial section is complete.

**Costing for hypothetical non-typhoidal salmonella outbreak, 2020, Nigeria**

Consultancies Costs

**Respondent details**

Name:

Position:

Email:

Phone:

Costing for hypothetical non-typhoidal salmonella outbreak, 2020, Nigeria

**Section 7 of 7 – Consultancies Costs**

*Please complete any monetary questions in* ***Nigerian Naira***

*If any section below is not applicable to the current outbreak, please write N/A*

*The following question is to be answered for each of the listed consultancy areas below (where applicable), and for any additional consultancy areas absent from the list:*

**1 - Total expenditure on consultancy used to support outbreak response activities**

1 Consultancy for database development

1 –

2 Consultancy for database management

1 –

3 Consultancy for data collection

1 –

4 Consultancy for data analysis

1 –

5 Consultancy for field epidemiology

1 –

6 Consultancy for biology/entomology

1 –

7 Consultancy for training

1 –

8 Consultancy for risk communications and media trainings

1 –

9 Consultancy on development of case management guidelines for safety hazards (zoonotic, food safety etc.)

1 –

Other 1 (specify)**: __________________________**

1 –

Other 2 (specify): **__________________________**

1 –

*Please identify any consultancy staff positions directly related to these outbreak investigation and response activities:*

1 Job title:

1 Number of individuals:

2 Job title:

2 Number of individuals:

3 Job title:

3 Number of individuals:

4 Job title:

4 Number of individuals:

NB - There is a more complex addition to this section, covering the percentage of total expenditure for each item reported and how it is distributed across each individual stage of the outbreak investigation. A percentage of total expenditure form for this section is to be completed after this initial section is complete.

**Costing for hypothetical non-typhoidal salmonella outbreak, 2020, Nigeria**

**Non-labour costs questionnaire: Percentage of total expenditure form**

**Respondent details**

Name: __________________________________________________

Institute & Position: __________________________________________________

Email: __________________________________________________

Phone: __________________________________________________

Date: ______/______/_________

**Instructions**

*Please complete the box below.*

*Each of the items listed by yourself in the previous* ***Office Materials Questionnaire*** *are listed below. For each item, please indicate below what* ***percentage*** *of total expenditure was incurred during each period of the outbreak. Periods of outbreak include:*

- ***Initial response period***
- ***Outbreak response period***
- ***Implementation, follow up & reporting period***

*Each box should contain a* ***single percentage****.*

*Each item row should total* ***100%***

| **ITEM** | **INITIAL RESPONSE PERIOD: *% of total expenditures***  Including the following activities:   - Prepare - Verify outbreak - Verify diagnosis - Construct case definition - Record cases - Perform descriptive epidemiology - Develop hypothesis - Evaluate hypothesis - Refine hypothesis - Reconcile evidence | **OUTBREAK RESPONSE PERIOD: *% of total expenditures***  Including:   - Implement control & prevention measures | **IMPLEMENTATION, FOLLOW UP AND REPORTING PERIOD: *% of total expenditures***  Including:   - Initiate or maintain surveillance - Disseminate findings | **TOTAL (%)**  (each individual row should total 100% - this is like the final questions) |
| --- | --- | --- | --- | --- |
|  |  |  |  |  |
|  |  |  |  |  |
|  |  |  |  |  |
|  |  |  |  |  |
|  |  |  |  |  |

Supplemental: Table of and basis for assumptions

| **S/No.** | **Assumptions and sources of Costs** | **Reference** |
| --- | --- | --- |
|  | Budget allocation – Labour (83.51%) versus non-labour (16.49%) Total attributable budget (Diarrhoeal Diseases Programme – Salmonellosis (1.3%): Health (Fed and States MoH) = N4,845,138,991; Agric (Fed and States MoA&RD) = N628,238,672.96 | Federal Ministry of Health (2017). Nigeria Health Financing Policy and Strategy, 2017. Available at: <https://nesgroup.org/download_policy_drafts/Nigeria-Health-Financing-Policy-Strategy_2017-21032019_1661875118.pdf>. Accessed 03 June 2023.  Chaitkin M. 2022. Intergovernmental Rivalry and Fragmentation: How Federalism Shapes Public Financial Management and Health Financing in Nigeria. Case Study Series on Devolution, Health Financing, and Public Financial Management. Washington, DC: ThinkWell. Available at: <https://thinkwell.global/wp-content/uploads/2022/04/Nigeria-Case-Study-April-2022.pdf>. Accessed 03 June 2023.  BudGIT, 2022. Appropriation Amendment: Federal Ministry of Agriculture And Rural Development, and National Veterinary Research Institute budgets. Available at: <https://budgit.org/wp-content/uploads/2023/01/2022-Appropritation-Bill.pdf>. Accessed 03 October 2023. Vanguard Newspaper, 2019. 2019: Buhari, 33 governors budget N15.737 trillion, available: <https://www.vanguardngr.com/2019/01/2019-buhari-33-governors-budget-n15-737-trillion/>. Accessed 3 October 2023. |
|  | Mean monthly salaries and time contributed to diarrhoeal disease programme | ***Field Survey***: Med lab: new intake N140,000, seniors 320,000; Pharmacists: new intake N130,000, seniors N300,000; Medical officers: new intake N240,000, seniors N620,000; Public Health Specialist N620,000; Epidemiologist Junior N240,000, Senior N620,000; Technicians N75,000; Veterinarian: new intake N240,000, seniors N325,000; Veterinary epidemiologist: new intake N240,000, seniors N480,000; Zonal Veterinary officers: state N500,000, Federal 490,000; Veterinary technician N85,000; Veterinary extension officer N240,000; Veterinary laboratory scientist N170,000; Project managers N780,000; Others: 60,000 – 150,000. Minimum wage for not technical officers: N30,000. |
|  | Office costs | Field survey |
|  | Purchase and distribution of resources | Resources (human and material) are purchased or distributed at different levels based on different considerations: National (n = 1), States (n = 36 plus FCT = 37), Regional (Zonal) (n = 109) and Local Government Authority (n = 774) |
|  | Vehicle price (N25 million) (Range: N6.93m – N25m) | Available at: <https://nigerianprice.com/prices-of-toyota-hilux-in-nigeria/> |
|  | Daily allowances to investigate an outbreak (limited) (N350,000 per outbreak) | Field survey |
|  | Communication costs | Based on co-contribution from the Diarrhoeal Programme |
|  | PPE Cost (US$ 13.04) | Bolas, T., Werner, K., Alkenbrack, S., Uribe, M. V., Wang, M., & Risko, N. (2023). The economic value of personal protective equipment for healthcare workers. PLOS global public health, 3(6), e0002043. <https://doi.org/10.1371/journal.pgph.0002043> |
|  | 100 pieces syringes with needles (N14,999.00) | Available at: <https://www.jumia.com.ng/mlp-syringes/> |
|  | Pipette tips (N14,091 per 1000 pieces) | Available at: <https://www.jumia.com.ng/generic-10ul-200ul-1000ul-5ml-pipette-micropipette-tip-for-123627222.html> |
|  | Laboratory support costs | Field survey |
|  | Poultry Fowl typhoid vaccines (NVRI) (N1000 per 100 doses) | Available at: <https://www.nvri.gov.ng/products>  Personal Communication: Dr. David Lazarus |
|  | Antibiotic therapy (Assuming that 50% of all human cases implement self-treatment at a cost of N13,309.10) | Field survey, Uzochukwu and Onwujekwe, 2004. |
|  | Cost of Widal’s test | Available at: <https://www.surjen.com/lab-test> |
|  | Hospitalization and Treatment costs (N22,815.60) | Field survey |
|  | Miscellaneous medical countermeasures costs (10% of cumulative) | Subject matters experts’ opinions |
|  | Consultancy costs (Equivalent of mean GHS support cost per country = US$1,000,000) | Subject matters experts’ opinions |
|  | Contingencies and Miscellaneous expenses (N100,000,000) | Subject matters experts’ opinions |

*Please note that a number of the non-labour costs are contributory services where many activities budget contribute to the Health budget pool, hence partial attribution in cost contribution was given to Non-Typhoidal Salmonellosis.*

##### **Supplementary Material 2**

Outbreak Costing Tool.

##### **Supplementary Table 3. Outbreak Costing Tool individual cost items for each non-labor cost category**

| **Office materials and equipment** | **Travel and transport** | **Communication** | **Laboratory support** | **Medical countermeasures** | **Consultancies** |
| --- | --- | --- | --- | --- | --- |
| Stationeries | Fuel costs | Airtime for national radio broadcasts | Personal protective equipment | Drugs for prevention: Vaccines | Database development |
| Printing/copies | Rented or hired vehicles | Airtime for national television broadcasts | Syringes | Antibiotic prophylaxis | Database management |
| Rented building space | Parking | Advertisements in national newspapers | Pipettes | Quarantine | Data collection |
| Rented equipment | Purchased vehicles | Airtime for local radio broadcasts | Reagents | Closing of food premises | Data analyses |
| Rented furniture | Maintenance and repair costs | Airtime for local television broadcasts | Shipment of materials | Animal culls | Field epidemiology |
| Internet | Lodging | Advertisements in local newspapers | Specimen collection | Disposal or decontamination of contaminated items | Biology/  entomology |
| Cellular data | Per diem expenses (e.g., food) | Wall poster advertisements | Specimen transport | Water chlorination | Training |
| Speciality software | Airfare for deployed personnel | T-shirts to raise outbreak awareness | Specimen processing | Impregnated bed nets | Risk communication and media trainings |
| Mobile phones | Taxi and bus fares |  | Identification of pathogens |  | Development of case management guidelines for safety hazards (e.g., zoonotic, food safety) |
| Solar panels to charge phones and computers |  |  | Data management |  |  |
| Global Positioning System devices |  |  | Data analysis and results |  |  |
| Mobile hotspots |  |  | Waste management |  |  |

##### **Supplementary Material 4**

Scenario Analysis.
